# A multilevel mixed effects analysis of informal carers health in Australia: the role of community participation, social support and trust at small area level

**DOI:** 10.1186/s12889-020-09874-0

**Published:** 2020-11-26

**Authors:** Itismita Mohanty, Theo Niyonsenga, Tom Cochrane, Debra Rickwood

**Affiliations:** 1 Health Research Institute, Faculty of Health, University of Canberra, Canberra, Australian Capital Territory 2617 Australia; 2 Psychology, Faculty of Health, University of Canberra, Canberra, Australian Capital Territory 2617 Australia; 3 Headspace National Youth Mental Health Foundation National Office, Melbourne, VIC Australia

**Keywords:** Informal carers health, Mental health, General health, Community participation, Social support, Trust, Small area, Multilevel mixed effects analysis

## Abstract

**Background:**

Informal carers suffer from worse health outcomes than non-carers due to their caregiving role. Yet, in a society carers health is as important as that of their care recipients. This study investigated the self-assessed mental and general health outcomes of informal carers in Australia. It evaluated the influence of carers’ personal social capital- a logically linked sequence of their social behaviour such as community participation, social support and trust in others- on their health outcomes. The study estimated the magnitude of small area level variation at Statistical Area Level 1 (SA1) along with individual level variation in carers’ health outcomes.

**Methods:**

The study used a multilevel mixed effects cross-sectional design using data from the Household Income and Labour Dynamics of Australia survey, wave 14. It included Australians aged 15 years and older that were surveyed in the year 2014. The sample consisted of 12,767 individuals and 5004 SA1s. The outcome measures included- mental health, general health and physical functioning, domains of the *Short Form 36 Questionnaire*, a widely used multi-dimensional measure of health-related quality of life.

**Results:**

Informal carers suffered from poor mental (Beta = − 0.587, *p* = 0.003) and general health (Beta = − 0.670, *p* = 0.001) outcomes compared to non-carers in Australia. These health outcomes exhibited significant variation acrossSA1s in Australia, with 12–13% variation in general and mental health. However, within small local areas, differences at the individual level, accounted for most of the variation in outcomes. Moreover, levels of community participation, personal social connection and trust, as perceived by individuals in the communities, had a positive influence on both mental and general health of carers and non-carers, and were more beneficial for carers compared to non-carers.

**Conclusion:**

It seems that the positive influence of social capital for carers helps them in coping with the negative impact of their caregiving duty on health outcomes. Findings suggested that some targeted community support programs for carers to build on their personal social cohesion and trust in their community could help in improving their poor health profiles. Moreover, improved informal carers’ health may help the health system in better managing their resources.

**Supplementary Information:**

The online version contains supplementary material available at 10.1186/s12889-020-09874-0.

## Background

The health and wellbeing of carers are considered as important as, and are essential to, the health and wellbeing of their care recipients. Yet, informal carers suffer from worse general and mental health outcomes than non-carers [1–3] Informal carers play a main role in providing care for someone, closely related to them, a friend or a neighbour, primarily in the home environment with a range of physical, mental and end-of-life health conditions, and disability [4]. They make a significant contribution to the care and wellbeing of people with a disability, mental illness, chronic condition, terminal illness and the elderly [5]. They are an integral part of both the community and the health system in any country.

Like elsewhere in the world, there is a growing demand for carers in the Australian formal health care sector [6]. At the same time, a low supply of informal carers presents an additional burden on Australia’s health and welfare [5]. This is likely to result in a strain on the health sector. In particular, poorer health outcomes are expected for those unable to afford formal care and those who prefer to stay home and be cared for by a family carer when they are sick or disabled or for palliative care [7]. An adverse impact on informal carers’ health due to their caregiving status, the complexities of the care requirement and/or the extended hours of caregiving, may further place greater pressure on the formal health care sector. In this scenario, it is hard to free informal carers of their caregiving responsibilities. This will add significant fiscal burden to government and society alike. Notwithstanding these challenges, it is likely that policy adjustments can be made to better support carers and lessen their burden of care, provide them with some level of relief and promote their health and wellbeing.

Previous research has found that a range of factors exert major influences on carers’ health, including their age, gender, socioeconomic status, relationship with the care recipient, the availability of a support network and their health behaviour [1, 3, 8, 9]. Some groups of carers suffer worse health outcome than non-carers, defined by their age and gender (women 50 years and above) and the health conditions of care recipients (Alzheimer’s disease/dementia/mental health) [10, 11]. Some Australian studies have found an additional burden on informal carers in rural areas because of inaccessibility of services and absence of social support services [2, 3]. A recent study found that marginalisation of informal carers in rural areas further exacerbates the impact of caregiving on their health [2]. These researchers found that caregiving can have cascading negative health effects when combined with reduced social interactions and geographical isolation as in rural and remote areas [2, 12]. An aspect that is often overlooked or has not been formally studied is the nature of the social environment in which the carer lives and interacts with. A thriving social environment may support carers to maintain a healthy life even if their caregiving proves to be burdensome. This includes the nature of the local community or local area in which the carer lives and the level of social capital experienced. Consequently, the role of carer’s community participation, social support and trust in their local community on their health is an important research topic that this study investigated. The study aimed to improve our understanding on how to create a flourishing local social environment for informal carers to facilitate healthy living with caregiving.

Social capital has been defined by Putnam et al. (1994) and paraphrased by Rocco et al. (2012) as “features of social organization, such as trust, norms, and networks that can improve the efficiency of society by facilitating coordinated actions” [13, 14]. Rocco et al. (2012), however focused their definition more on social connectedness rather than the simple existence of social institutions that strengthen and sustain the society [14]. They present both social connection and social network as major components of social capital [14]. Putnam et al. (1994), on the other hand, highlighted the health promoting role of social capital in their study, and defined social capital as “a catalyst of coordination/cooperation, an essential device to achieve better (social and/or economic) outcomes” [13, 14]. Likewise, as Rocco et al. (2012) reported, trust is promoted as a determinant of social connections, therefore, an indicator of social capital, given its role in initiating and maintaining social interactions [14–16].

Previous studies have highlighted the positive influence of social capital on health of the general population [14, 17] but, few studies have really investigated the role of social capital on informal carers’ health and how it interacts with their caregiving responsibility. For example, the extent to which these social capital-health relationships vary by carer and non-carer status is worth investigating. Additionally, the extent to which these relationships vary by small regional areas in which the carers live warrant more research. Thiel (2016) analysed the role of social capital in buffering the negative relationship between informal care provision and mental health in German and Oshio and Kan (2016) in Japan [18, 19]. They found that stronger social ties moderate the negative association between caregiving and carers’ mental wellbeing. Thiel (2016) found that the protective role of social capital was particularly strong for caregivers with high time commitments. Thiel (2016) also highlighted that how we capture the social capital in studies is important, as he found that the moderating role of social activities can neither be explained by the caregivers observed characteristics correlated with social capital, nor by features of the caregiving process. Likewise, using data from the European Social Survey for 14 European countries, Rocco et al. (2012) studied how individual and/or community-level social capital positively affects health in the general population and addressed the challenge of assessing causality in the relationship between social capital and health. In their view, community social capital (defined at regional level) appears not to affect health once individual-level social capital is controlled for [14]. Previous methodological studies, Berry et al (2010) and Berry and Rickwood (2000), emphasized the role of measuring community social capital at the individual level and their influence on general and mental health [20, 21]. These researchers highlighted that social capital is associated with better health, but it is important to understand different components of social capital and their underlying construct and how these components are formed and associated with different types of health: general, mental and physical [20]. Overall, individual or personal social capital is particularly important [14, 22]. Berry et al. (2000) note that this is how the nature of the local community and community social capital are experienced at the individual level. In their view, social capital (a societal level construct) can be measured at an individual level and is called personal social capital [21]. Understanding how indicators of personal social capital are associated with carer/non-carer health, how they moderate the effect of caregiving on carer health and the magnitude of regional variation of carer’s health outcomes are important contributions of this research.

In Australian Statistical Geographical Standard, SA1s (Statistical Area Level 1) are the smallest geographical areas with an average population around 400 people. SA1s are broadly based on the concept of a functional neighbourhood area, that is, the area within which many people commute or travel to access services [23]. SA1s are designed to be either a predominantly rural or urban in character, generally having a lower population in rural and remote areas than in urban areas. SA1s are expected to be functionally based on more homogenous population than the other higher level of spatial units [23]. In this study we accounted for SA1 level variations in carer health outcomes. Therefore, along with individual and household confounders and personal social capital variables, we have considered SA1s as the next higher level of spatial geography in a mixed effects hierarchical model as community participation, social connection and trust and reciprocity at this level might influence informal carers’ health.

### Research question and hypothesis

It is recognised that overall informal cares suffer from worse health outcomes compared to non-cares [1–3]; this study investigated an important question that if these disparities could be moderated through carers’ social capital. For example, the study investigated if the nature of the local community and community social capital experienced at the personal level moderated carer’s health. The study is based on the hypothesis that social capital plays a significant role in informal carers health where high level of community social capital experienced at the personal level could help in overcoming the health disadvantage that they face due to the caregiving responsibility. This provides information for policy intervention, for example, which aspects of the community social capital/environment could be targeted to improve carers’ health. The study used a large nationally representative household survey sample data to study carers’ health in terms of their general, physical and mental health compared with non-carers, and investigated the impact of their personal social capital, while controlling for variations at the local area, SA1 level and other confounding factors. Using a multilevel mixed effects study design, allowing for SA1 level variations in the initial (mean) values of general/mental/physical health components scores and in the nature of the relationship between caregiving status and health outcomes, the study analysed the extent to which individual level appraisal of community-social capital indicators, expressed in specific components, influenced self-assessed mental and general health of carers compared to non-carers (effect modification or moderation effect). This provides a foundation for designing community level social support programs for carers to improve their health outcomes.

## Methods

### Data source

This study used data from the Household Income and Labour Dynamics of Australia (HILDA) survey, a major large-scale population-based longitudinal survey of Australian households available over a period of 17 years (2001–2017). HILDA survey follows the lives of members of sampled households and collects, at each wave, information on their economic and personal well-being, labour market dynamics, family life, household and family relationships, income and employment, health and education [24]. We analysed wave 14 data, the latest available, as information on community participation, social connection, trust and reciprocity were not available in each wave. Our sample contained Australians aged 15 years and over who had completed the self-completion questionnaire (SCQ) as most of our dependent/independent variables come from the SCQ. For more information on HILDA survey sampling methodology please refer to HILDA User Manual and HILDA project discussion paper series #1/15 [24, 25]. Our sample consisted of 12,767 individuals located over 5004 SA1s.

### Measures

#### Statistical area level

The geographical units of analysis in this study were Australian Census of Population and Housing (ABS, 2011) Statistical Area Level 1 (SA1). SA1s have generally been designed as the smallest local or neighbourhood units for the release of census data. SA1s have a population of between 200 and 800 people with an average population size of approximately 400. Whole SA1s aggregate to form Statistical Areas Level 2 (SA2) in the Australian Statistical Geography Standard (ASGS) Main Structure [23].

#### Health outcomes

Following Mohanty and Niyonsenga (2019), [1] the health outcome variables were self-reported health scores from a widely used multi-dimensional instrument measuring the health-related quality of life, the *Short Form 36 Questionnaire* (SF-36), reported in HILDA surveys [26]. We chose the general health, physical functioning and mental health domains as our outcome variables. Component scores of the SF-36 domains are standardised and range from 0 to 100, higher scores indicating better health [26]. Note that these SF-36 components are generic measures of health, as opposed to the ones that would target specific age, disease, or treatment groups [1].

#### Socio-economic and demographic control variables

The study involved a set of explanatory variables on the community and societal social capital realised at individual level, socio-economic and demographic variables, physical activity status, any major adverse health event that occurred in the previous year, that may have influenced the general, physical or mental health of an individual along with his/her care-giving status. Like in Mohanty and Niyonsenga (2019), the primary independent variable, the care-giving status, is a dichotomous variable coded as 1 if the individual actively cares for a household member/non-resident due to a long-term health condition or elderly status and 0 otherwise [1]. The study also controlled for the number of hours a person spends on care-giving duties in a week irrespective of their carer status. Tables A1-A3 (Additional file 1) present the indicators of community participation, social support and trust-reciprocity respectively, while Table 1 lists the predictors and outcome variables used in this analysis, along with their descriptive statistics. All these variables included in the model are fully detailed in the 2017 HILDA User Manual – Release 16 [24].

**Table 1:** Descriptive Statistics

Variable	Mean/Percent (*N* = 12,767)	Standard. Deviation	Minimum Value	Maximum Value
SF36 General Health Component Score	67.62	20.83	0.00	100
SF36 Mental Health Component Score	74.05	17.58	0.00	100
Actively cares for a household member/non-resident due to long-term health condition, elderly (%)	7.76%	0.27		
Time spent in (hrs/mins) per week Caring for disabled/elderly relative (*n* = 8416)	1.69	9.87	0.00	168
Age	45.10	18.71	15.00	97
Gender: 1 for male	47%	0.50		
Household financial year gross total income ($)	124,708	111,903	−74,269	2,827,827
Life events in past year: Serious personal injury/illness	10%	0.30		
Educational level				
Tertiary University Education (Base Category)	26.44%	0.44		
Avd diploma, diploma, Cert III or IV	31.71%	0.47		
Year 12	15.15%	0.36		
Year 11 and below	26.71%	0.44		
Employment Status				
Employed - works 35h hours a week (Base Category)	40.89%	0.49		
Employed - works less than 35 h a week	21.23%	0.41		
Unemployed, retired, home duties, students & others	37.87%	0.49		
How often participate in Physical Activities?				
Not at all (Base Category)	10.95%	0.31		
Less than once a week	16.68%	0.37		
1 to 2 times a week	23.54%	0.42		
3 times a week	15.75%	0.36		
More than 3 times a week	33.09%	0.47		
Social Capital Summary Variables				
Civic engagement, political participation and breadth of participation	1.93	1.13	−0.13	7.17
Informal social connectedness	5.69	1.20	1.20	8.87
Personal social exclusion	5.78	1.64	1.47	12.12
Personal social cohesion	8.97	1.29	1.79	12.56
Level of trust in the community	7.66	1.19	1.61	11.28
Level of distrust in the community	6.98	1.56	1.78	12.44

Note: The Table 1 presents summary statics of the variables used in the fully-adjusted multi-level mixed effects models for the SF36 mental and general health outcomes in wave 14 of HILDA data

#### Personal social capital variables

Following Berry et al (2010) and Berry and Rickwood (2000) [20, 21], we identified 12 variables in HILDA that assessed community participation, 10 variables that described informal social connectedness or exclusion and 7 variables that described level of trust/distrust in the community. We conducted principal component factor analysis to summarize and identify any common underlying theme in each category separately. Our analysis clearly identified two different underlying constructs or factors for each of the community participation, personal social cohesion and trust and reciprocity categories. Following Berry et al. (2010), [20] we named Factor 1 as ‘Civic engagement, political participation and breadth of participation’ and Factor 2 as ‘Informal social connectedness’ in the community participation category. Likewise, in the personal social cohesion category, the two factors were named as ‘Personal social exclusion’ and ‘Personal social cohesion’. Further, in the trust and reciprocity category, the factors were named as ‘trust’ and ‘distrust’. The set of variables grouped under these factors in each category, their rotated factor loadings (pattern matrix), unique variances are presented in Tables A1-A3 in the Additional file (Additional file 1).

### Statistical methodology

The study used cross-sectional multilevel mixed effects regression analysis considering the hierarchical or clustered structure of the data: individuals/households are clustered within geographical areas or communities they live; and communities are also nested within other higher administrative units [27–29]. Communities are more likely to share similar policy and health care infrastructure than those living in other communities.

Our explanatory variables were grouped into two levels to reflect the hierarchical nature of the data. Level 1 variables corresponded to individual level characteristics. At the individual level, the model included carer/non-carer status, time spent caring for disabled/elderly relatives, employment status, age, gender, any major injury/illness in past year, educational level, physical activity status, household gross total income and the sets of community participation, social connection, trust and reciprocity summary variables as main effects. To test the moderation effect of the community participation, social connection and trust and reciprocity on carer health, the model also included interaction-effects of carer’s status with these social capital summary variables. Level 2 variables corresponded to SA1 random effect (i.e., allowing for variations in average values of health scores across SA1s) and caregiving status (carer/non-carer) as a random slope (i.e., allowing for variations in the impact of caregiving on carers’ health across SA1s).

We fitted separate models for each of the SF-36 component scores namely, General Health, Mental Health and Physical Functioning, as outcome measures. We estimated a series of regression models on each health outcome measure (7 models each) and added the explanatory variables sequentially in groups into the successive models. Our analysis for the SF-36 physical functioning measure did not show any significant effects of carer/non-carer status and the results are not presented here. Analyses were performed using STATA 14 (XTMIXED FE || RE:) with robust standard errors estimation [30, 31].

The model 1 in each health component score (Tables 2 and 3, first column), was the simplest multilevel model with one level 1 explanatory variable - carer/non-carer status (main effect), and SA1 level random effects (intercept) at level 2.

**Table 2:** Multilevel mixed effects estimates for SF36 general health component

Variables (no. of observations = 12,767 and no. of SA1s = 5004)	Model 1: Coeff (95% CI)	Model 2: Coeff (95% CI)	Model 3: Coeff (95% CI)	Model 4: Coeff (95% CI)	Model 5: Coeff (95% CI)	Model 6: Coeff (95% CI)	Model 7: Coeff (95% CI)
Variance (Individual-level random effects)	381.874***(370.388, 393.716)	376.868*** (365.081, 389.037)	363.916*** (352.640, 375.552)	340.609*** (330.159, 351.389)	335.010*** (324.729, 345.634)	281.753*** (273.349, 290.414)	281.744*** (273.339, 290.408)
Variance (SA1 level random effect)	51.784***(42.917, 62.483)	54.451***(44.969, 65.931)	43.959*** (35.467, 54.483)	31.729*** (24.525, 41.048)	32.350*** (25.171, 41.577)	13.973*** (9.359, 20.863)	13.960*** (9.346, 20.852)
Variance (Carer random slope)		67.163** (32.154, 140.29)	54.575** (23.572126.355)	36.886* (12.601, 107.967)	33.125* (10.240, 107.160)	37.124** (14.716, 93.652)	37.268** (14.7694, 94.042)
Covariance (Carer and SA1)		−17.374 (−38.073, 3.324)					
Carer	−5.045*** (−6.387, −3.704)	− 5.336 *** (−6.773, − 3.899)	−5.504*** (−6.88, −4.125)	−4.302*** (− 5.614, −2.992)	−4.264*** (− 5.555, − 2.973)	− 1.135* (− 2.459, 0.189)	−2.565 (− 14.750, 9.621)
Civic engagement, political participation and breadth of participation			0.071 (− 0.242, 0.385)	−0.098 (− 0.398, 0.202)	−0.302** (− 0.602, − 0.003)	−.002 (− 0.279, 0.276)	0.009 (− 0.279, 0 .297)
Informal social connectedness			3.913*** (3.622, 4.205)	1.034*** (0.706, 1.362)	0.628*** (0.297, 0.959)	0.545*** (0.239, 0.851)	0.547*** (0.230, 0.865)
Personal social exclusion				−3.447*** (− 3.671, − 3.224)	− 2.894*** (− 3.130, − 2.657)	− 1.988*** (− 2.205, − 1.771)	− 1.995*** (− 2.221, − 1.769)
Personal social cohesion				3.059*** (2.774, 3.343)	2.599*** (2.308, 2.890)	1.972*** (1.706, 2.237)	2.009***(1.732, 2.286)
Trust					1.753*** (1.442, 2.063)	2.416*** (2.125, 2.708)	2.348*** (2.045, 2.651)
Distrust					−1.170*** (− 1.401, −0.940)	− 1.421*** (− 1.632, − 1.211)	−1.409*** (− 1.629, − 1.191)
Carer # Civic engagement, political participation and breadth of participation							− 0.181 (−1.177, 0.815)
Carer # Informal social connectedness							0.467 (−0.637, 1.570)
Carer # Personal social exclusion							−1.886*** (−2.647, − 1.125)
Carer # Personal social cohesion							1.601*** (0 .680, 2.523)
Carer # Trust							3.207*** (2.197, 4.217)
Carer # Distrust							−1.589*** (−2.335, −0.843)

***Notes.*** All the *p*-values have been replaced by stars and categorised as follows. ***: *p* < 0.01; **: *p* < 0.05; *: *p* < 0.10; However, confidence intervals use the usual 95% confidence level. Model 1: Null model with SA1 random effects and carer status at individual level; Model 2: Model 1 added with carer random slopes at SA1 level; Model 3: Model 2 added with community participation at individual level; Model 4: Model 3 added with personal social connection at individual level; Model 5: Model 4 added with trust at individual level; Model 6: Model 5 added with other individual level confounders; Model 7: Model 6 with community and social connection interactions with Carer

**Table 3:** Multilevel mixed effects estimates for SF36 mental health component

Variables (no. of observations = 12,767 and no. of SA1s = 5004)	Model 1 Coeff (95% CI)	Model 2 Coeff (95% CI)	Model 3 Coeff (95% CI)	Model 4 Coeff (95% CI)	Model 5 Coeff (95% CI)	Model 6 Coeff (95% CI)	Model 7 Coeff (95% CI)
Variance (Individual-level random effects)	270.740*** (262.516, 279.222)	267.462*** (259.074, 276.121)	245.992*** (238.372, 253.855)	202.326*** (196.193, 208.652)	335.019*** (324.729, 345.634)	183.028*** (177.458, 188.773)	183.003*** (177.434, 188.748)
Variance (SA1 level random effect)	39.162*** (32.587, 47.063)	38.244*** (31.498, 46.434)	25.019*** (19.585, 31.959)	15.656*** (11.755, 20.851)	32.350*** (25.171, 41.577)	12.569*** (9.127, 17.309)	12.556*** (9.116, 17.294)
Variance (Carer random slope)		47.688** (23.183, 98.096)	37.779** (16.590, 86.032)	44.053*** (24.043, 80.719)	33.125**(10.240, 107.160)	28.850** (12.680, 65.640)	28.255** (12.210, 65.384)
Covariance (Carer and SA1)		4.101 (−10.91, 19.114)					
Carer	−3.863*** (−4.996, −2.730)	−3.890*** (−5.145, − 2.635)	−4.164*** (−5.335, − 2.994)	−2.626*** (− 3.712, − 1.540)	− 4.264*** (− 5.555, − 2.973)	−1.770*** (− 2.879, −0.661)	1.381 (−8.751, 11.513)
Civic engagement, political participation and breadth of participation			0.827*** (0.570, 1.084)	0.630*** (0.399, 0.861)	−0.302** (− 0.602, − 0.003)	.082 (− 0.144, 0.308)	.097 (−.137, 0.331)
Informal social connectedness			4.691*** (4.452, 4.930)	1.279*** (1.026, 1.531)	0.628*** (0 .297, 0.959)	1.039*** (0 .790, 1.288)	1.035*** (0.777, 1.293)
Personal social exclusion				−4.538*** (− 4.710, − 4.366)	−2.894*** (− 3.130, − 2.657)	− 3.656*** (− 3.832, − 3.479)	−3.669*** (− 3.852, − 3.486)
Personal social cohesion				3.063*** (2.844, 3.282)	2.599*** (2.308, 2.890)	2.600*** (2.384, 2.817)	2.659*** (2.434, 2.885)
Trust					1.753*** (1.442, 2.063)	1.849*** (1.612, 2.086)	1.823*** (1.577, 2.069)
Distrust					−1.170*** (− 1.401, −.940)	− 1.310*** (− 1.482, − 1.139)	− 1.312*** (− 1.489, − 0.134)
Carer # Civic engagement, political participation and breadth of participation							−0.091 (− 0.922, 0.740)
Carer # Informal social connectedness							1.126** (0.207, 2.046)
Carer # Personal social exclusion							−3.456*** (−4.091, −2.820)
Carer # Personal social cohesion							1.912*** (1.143, 2.681)
Carer # Trust							2.133*** (1.291, 2.976)
Carer # Distrust							−1.343*** (−1.964, −0.721)

***Notes.*** All the *p*-values have been replaced by stars and categorised as follows. ***: *p* < 0.01; **: *p* < 0.05; *: *p* < 0.10; However, confidence intervals use the usual 95% confidence level. Model 1: Null model with SA1 random effects and carer status at individual level; Model 2: Model 1 added with carer random slopes at SA1 level; Model 3: Model 2 added with community participation at individual level; Model 4: Model 3 added with personal social connection at individual level; Model 5: Model 4 added with trust at individual level; Model 6: Model 5 added with other individual level confounders; Model 7: Model 6 with community and social connection interactions with Carer

In model 2 for each health component score (Tables 2 and 3, second column), we additionally included carer/non-carer status as a random slope at level 2 - SA1 level. This means that we also allowed the nature of the relationship between the carer status and the health component scores to vary across SA1s (random slope) [32, 33]. In models 3–5 (Tables 2 and 3, 3^rd^-5th column), we added the summary variables for community participation, personal social connection and trust and reciprocity in sequence. We added the other individual level confounders such as: age, gender, employment, education, household income, any major personal injury/illness last year and the physical activity/exercise status in models 6. Finally, in models 7 (Tables 2 and 3, 7th column), we interacted the community participation, social connection and trust and reciprocity level summary variables with carer status.

Estimates of effects were reported with associated 95% confidence intervals as suggested by NEJM guidelines [34]. The a priori level of significance was set at the usual 5% alpha and all *p*-values reported in Tables using the asterisk convention: ***: *p* < 0.01; **: *p* < 0.05; *: *p* < 0.10, [35] with the last category meant to show that a “trend towards statistical significance” has to be noted [36–38].

## Results

The series of multilevel mixed effects’ estimates for the SF36 health component scores are presented in Tables 2 (general health) and 3 (mental health) respectively. The first four rows in Tables 2 and 3 present: the variance components (i.e., residual variance or unexplained individual level variation; SA1 level variations in the initial/average health scores (random effects); SA1 level variations in the nature of relationship between carer status and carer health (Carer random slopes); SA1 level covariance between carer status and carer’s health; followed by the individual level fixed effects’ estimates with associated 95% confidence intervals (95%CI).

### Carer health and small area differences

#### General health

**Fig. 1:**
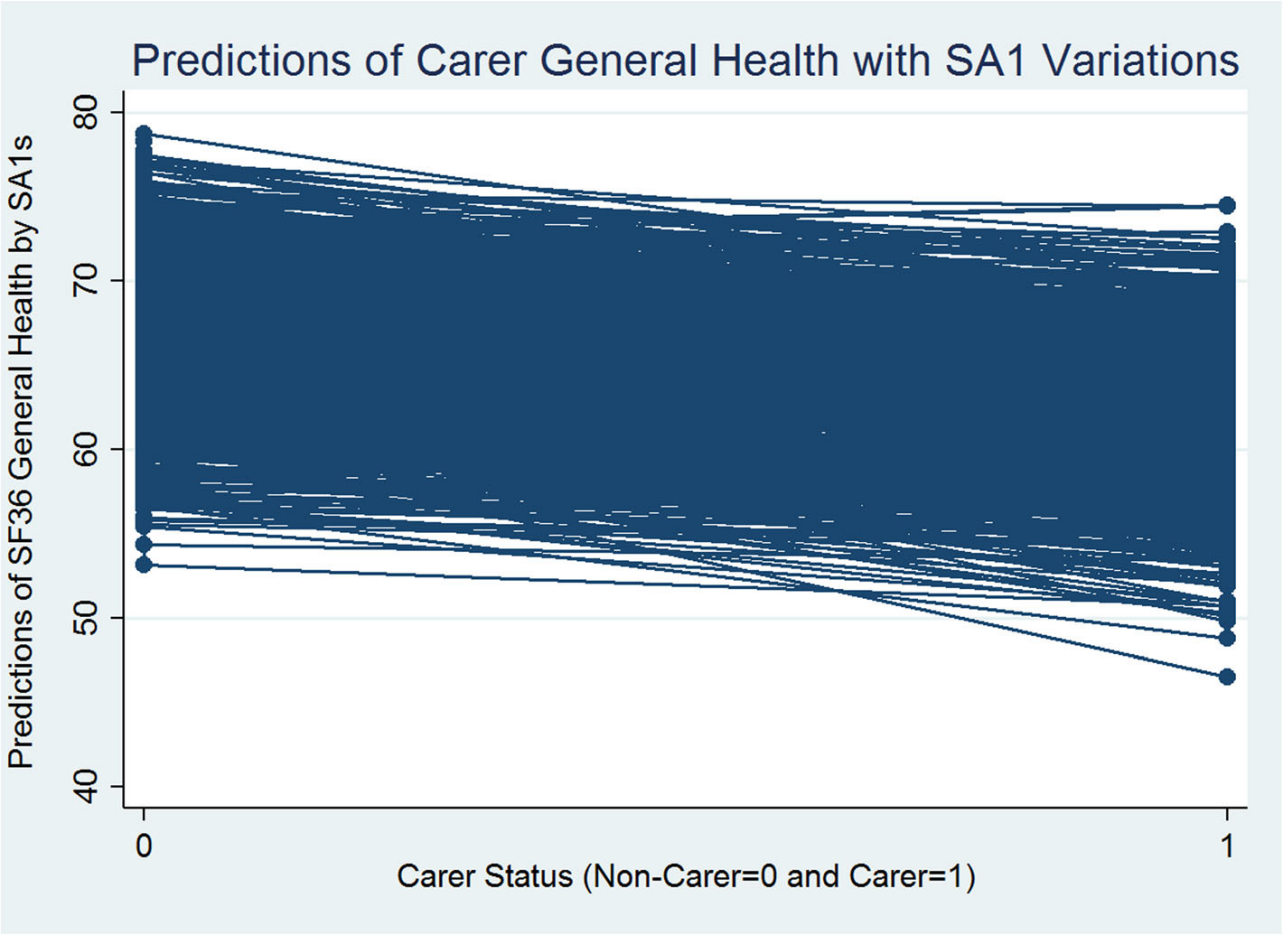
Carer/Non-Carer General Health and Statistical Area Level 1

The Table 2 (1st Column) presents between individual (within SA1) and between SA1 (level 2) variations in general health. It is estimated that between SA1 variance is 51.784, and the within-SA1 between-individual (level 1) variance is 381.874, leading to the total variance of 433.658 (51.784 + 381.874). Thus, the variance partition coefficient at SA1 level (VPC) is 51.784/433.658 = 0.119, which indicates that nearly 12% of the variation in general health can be attributed to differences between small areas at SA1 level. The rest, 88% of the variation, is between individuals within SA1s. Individual level carer status has a statistically significant negative influence (− 5.045 points-difference). Figure 1 presents the predicted values of carer and non-carer general health with SA1 variations estimated from Model 1.

**Fig. 2:**
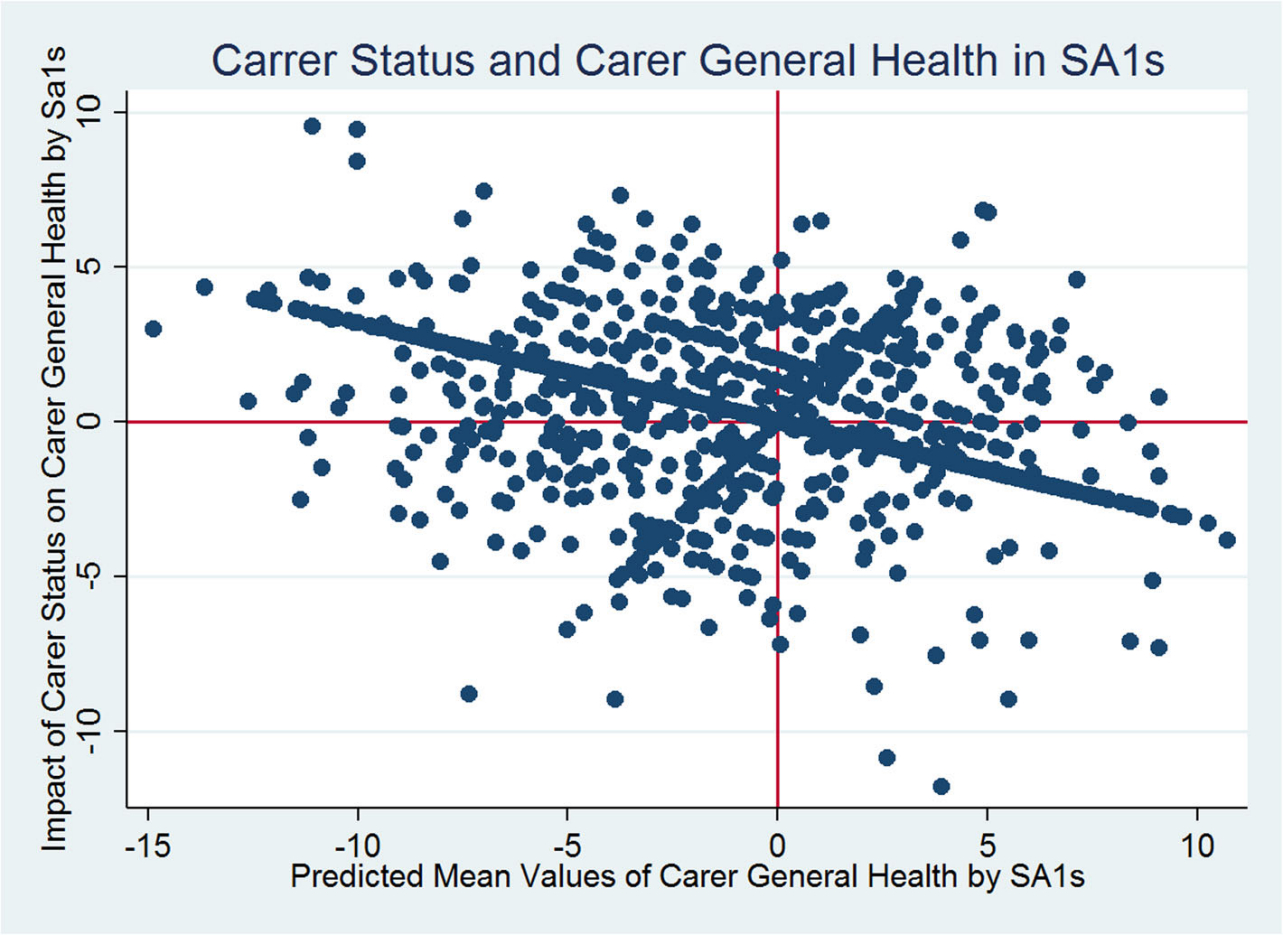
Impact of Caregiving on General Health by Statistical Area Level 1

Figure 1 reveals that generally there are statistically significant negative differences between the carer and non-carer predicted general health scores across SA1s. Carers’ predicted general health scores are significantly smaller than non-carers. The random slope for carer/non-carer status (i.e., SA1 level variations in caregiving effects) introduced in models 2 (Table 2) reveals statistically significant between SA1-carer-status-effects for general health. It means that the nature and the level of impact of caregiving on general health vary across SA1s and this variation in impact is statistically significant. Figure 2 presents a scatterplot of the SA1 level predicted mean general health scores (x-axis: SA1 random intercepts) and the extent of variability between the effects of caregiving status on general health (y-axis: SA1 carer’s status random slopes) estimated from model 2. Each dot point on the scatterplot represents an SA1.

Figure 2 reveals a general pattern of negative covariation (although non-significant) between intercepts (mean values) and slopes (changes), with SA1s with smaller intercepts having larger slopes. For example, SA1s in the top-left quadrant had a lower-than-average general health scores but a more-than-average influence of caregiving status on general health. Then again, SA1s in the bottom-left quadrant had a below-average mean scores, also a below-average influence of caregiving status on general health. However, the variability estimates from models 2–7 indicate that as we improve the model specifications sequentially adjusting for other covariates, the between-SA1 differences decrease from 13% in model 2 to less than 5% in the fully adjusted models 6 and 7.

#### Mental health

**Fig. 3:**
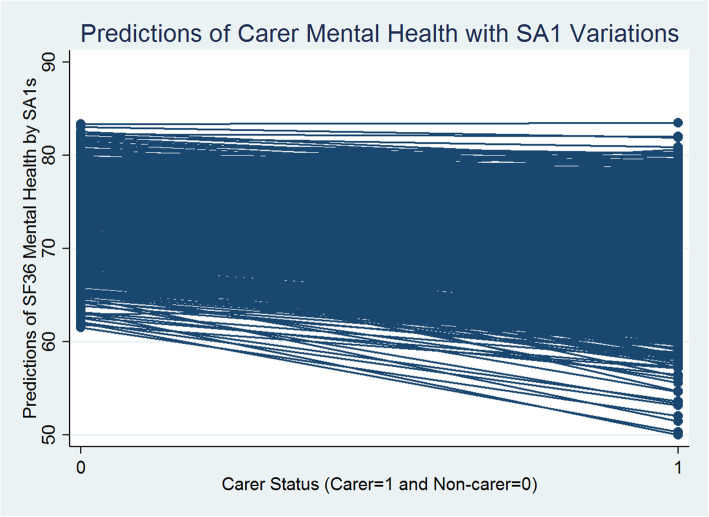
Carer/Non-Carer Mental Health and Statistical Area Level 1

The Table 3 (1st Column) presents a similar story. The between-SA1 (level 2) variation in mental health is estimated as 39.162, and the within-SA1 between-individual (level 1) variation is estimated as 270.74, leading to the total variance of 309.902 (39.162 + 270.74). Thus, the VPC at SA1 level is 39.162/309.902 = 0.126, which indicates that nearly 13% of the variation in mental health can be attributed to differences between SA1s and the rest, 87% of the variance, is between individuals within SA1s, with individual level carer status having a statistically significant negative influence on mental health (− 3.863 points-difference). Figure 3 presents the predicted values of carer and non-carer mental health with SA1 variations estimated from Model 1.

**Fig. 4:**
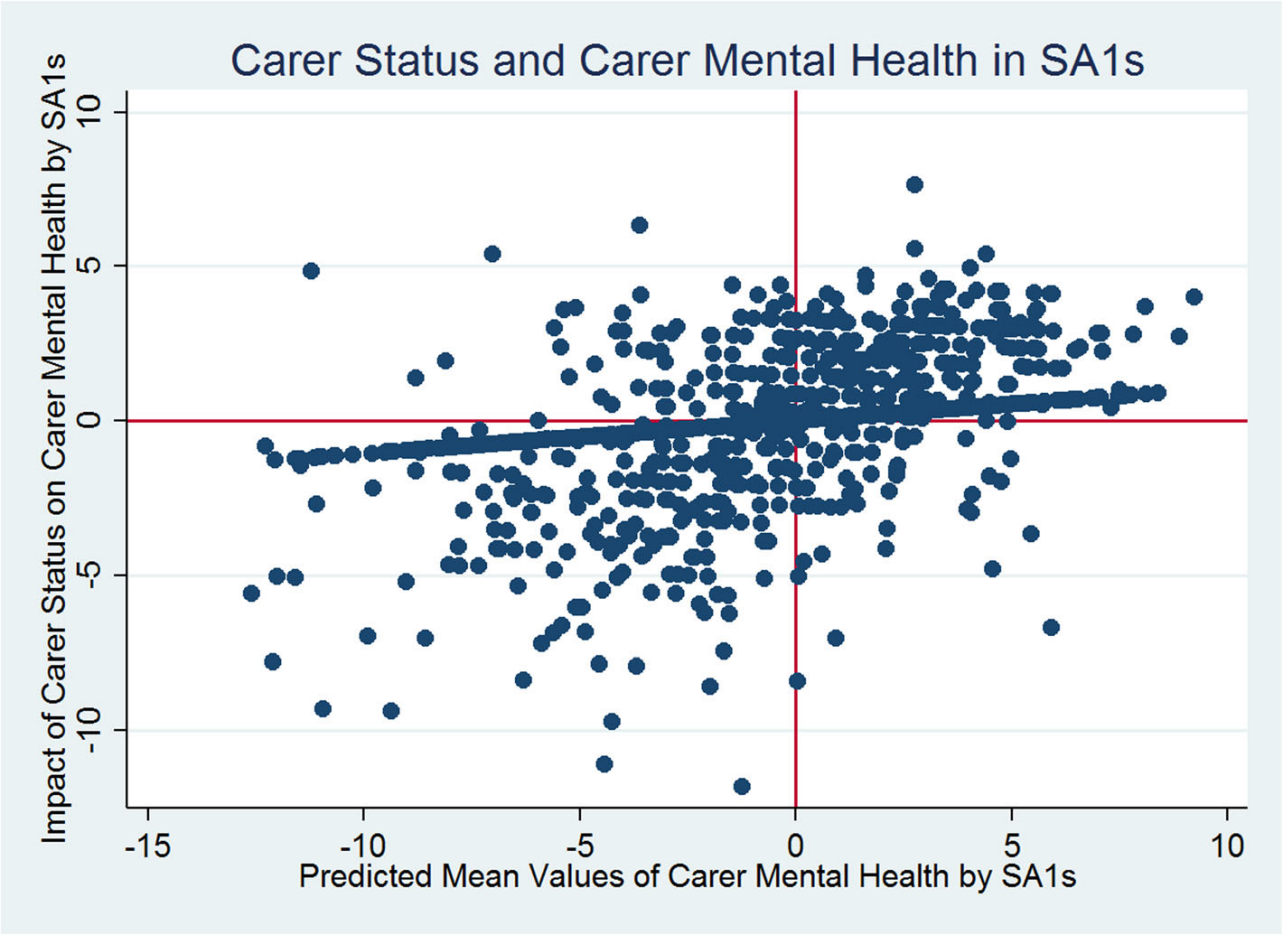
Impact of Caregiving on Mental Health by Statistical Area Level 1

Figure 3 reveals that generally there are statistically significant negative differences between the carer and non-carer predicted mental health scores across SA1s. Carers’ predicted mental health scores are significantly smaller than non-carers on average and these differences vary systematically across SA1s. The random slope for carer/non-carer status (i.e., SA1 level variations in caregiving effects) introduced in models 2 (Tables 3) reveals statistically significant between SA1-carer-status-effects for mental health. It means that the nature and level of impact of caregiving status on mental health vary across SA1s and this variation in effects is statistically significant. Figure 4 presents a scatterplot of the SA1 level predicted mean mental health scores (x-axis: SA1 random intercepts) and the extent of variability between the effects of caregiving status and mental health (y-axis: SA1 carers’ status random slopes) estimated from model 2. Each dot point on the scatterplot represents an SA1.

Figure 4 reveals a pattern of positive covariation (although too weak to be statistically significant) between intercepts (mean values) and slopes (changes), with SA1s with smaller intercepts having smaller slopes, and SA1s with larger intercepts having larger slopes. For example, SA1s in the top-right quadrant had higher-than-average mental health scores and more-than-average influence of caregiving on mental health. On the other hand, SA1s in the bottom-left quadrant had below-average mean scores, also below-average influence of caregiving on mental health. Like the general health, for mental health estimation in Table 3, the variability estimates from models 2–7, indicate that as we improve the model specifications sequentially adjusting for other covariates, the between-SA1 differences decrease from 13 to 6% in the fully adjusted models 6 and 7.

### Carer and non-carer health

**Fig. 5:**
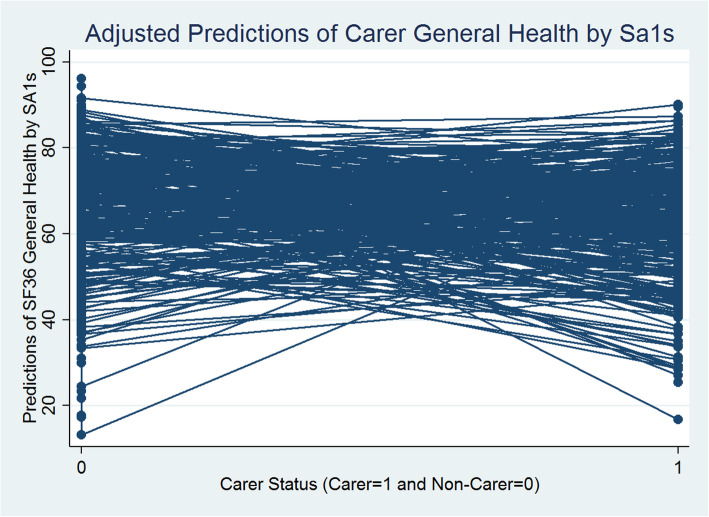
Carer/Non-Carer General Health Adjusted for Social Capital, Other Confounders and Social Capital Interacted with Carers by Statistical Area Level 1

**Fig. 6:**
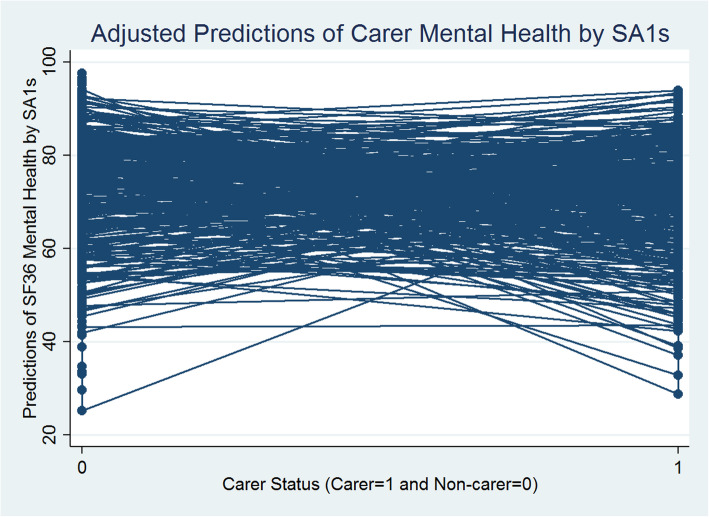
Carer/Non-Carer Mental Health Adjusted for Social Capital, Other Confounders and Social Capital Interacted with Carers by Statistical Area Level 1

Overall, carers show significant disadvantages in both mental and general health outcomes in models 1–6 (Tables 2 and 3; Figs. 1 and 3). These carers’ disadvantages remain highly statistically significant (at 1% significance level) even after controlling for a range of social capital and other individual level social, economic, demographic and physical activity characteristics, in the models 3–6 (Tables 2 and 3). Carers report worse health scores that are on average 4 points less than the non-carers for mental health and 5 points less for general health. However, the magnitude of the impact of caregiving on general and mental health drastically declines by almost 3 points for both the health component scores (in model 6 in Tables 2 and 3) and becomes statistically non-significant when we introduce the interactions between carer status and social capital variables (in model 7 in Tables 2 and 3; Figs. 5 and 6). In summary, the magnitude of the impact of caregiving decreased as we added more covariates to explain individual level variation in both the health components. Figures 5 and 6 present the adjusted predictions of general and mental health mean values (estimated from models 7) across SA1s respectively, that reveal almost similar ranges for predicted scores for carers and non-carers, with a mixture of increasing and decreasing effects of caregiving status. It could be argued that carers’ social capital has a statistically significant moderation effect that almost completely compensates for the negative impact of caregiving on health outcomes.

### Social capital and Carer health

#### Community participation

Individual levels of civic engagement, political participation and breadth of participation exerted positive and statistically significant influences on mental health (Table 3) in models 3 and 4 only. They did not show any consistent patterns in the fully adjusted models 6 and 7.

An individual’s informal social connectedness showed a positive and statistically significant relationship with both the general (Table 2) and mental health (Table 3) status even though with decreasing coefficients as we improve the model-specification between models 1–7. In the fully adjusted interaction model 7, informal social connectedness no longer remained statistically significant for carers’ general health, even if it continued to remain statistically significant for non-carers (Table 2). Nevertheless, informal social connectedness remained statistically significant for both carers’ and non-carers’ mental health in the interaction model 7 (with a bigger coefficient for carers) in Table 3.

#### Personal social cohesion

The variables representing individuals’ personal social cohesion and exclusion exerted statistically significant influence on both general and mental health in the expected directions (models 4–7 in both Tables 2 and 3). These two variables remained statistically significant in the fully adjusted interaction model 7 for both carers’ and non-carers’ general and mental health, with significant carer compared with non-carer differences (significant effect modification).

#### Trust and reciprocity

The variables representing individuals’ level of trust or distrust in the community exerted statistically significant influence on general health and mental health with expected signs. Further, the influence of these variables increased (with increasing coefficients) as we improved the specification in models 6 and 7, with stronger influence for carers (than non-carers) in model 7, and significant carer compared with non-carer differences (significant effect modification).

## Discussion

This paper presents a general population-based multi-level mixed effects analysis of individual level self-assessed mental and general health status of Australians in relation to their informal caregiving status. The study investigated the moderating effects of social capital indicators on carer/non-carer health outcomes. Additionally, the study accounted for the small area level variation in the carer/non-carer health outcomes, by allowing for variation in the average values of the health component scores as well as variation in the type of relationship between carer status and health by SA1s. The analysis supports evidence that informal carers do suffer from worse health than non-carers both in terms of their general and mental health. There are statistically significant small area level variations in health status both in their overall mean values as well as in the extent to which carer status influences health outcomes. The study adds significant value to the caregiving and health literature for its findings on the moderating effects of individuals’ social capital on their health outcomes. Carers’ social capital proves to be important for counteracting the negative health effects of caregiving on health. Additionally, the study yields significant inferences for SA1 level policy perspective to improve overall general and mental health status of carers and reduce the impact of caregiving on health.

### Carer and non-carer health

Carers in general suffered from worse health outcomes than non-carers, with values almost 5 points lower in magnitude in general health and 3.8 points lower in mental health. The inverse relationship between caregiving and health remained statistically significant as we introduced other confounding individual level characteristics such as their socio-demographic, economic and health behaviour, along with the social capital variables one-by-one in separate models. In the penultimate model (Model 6), the carer status continued to remain negatively significant with a much lower magnitude (coefficient of − 1.1 in general health and − 1.7 in mental health). These findings are aligned with previous studies which highlighted the extent to which caregiving was associated with poorer health status [1, 19, 39]. Oshio and Kan (2016) [19] looked specifically into the effect of informal caregiving on caregivers’ mental health and confirmed the same adverse impact as found in previous studies (e.g., Cameron et al., 2008; Binder & Freytag, 2013) [40, 41]. Stacey et al. (2018), [42] revealed a similar negative effect of caregiving on other chronic conditions, such as asthma and diabetes, which may in turn impair an individual’s general and mental health.

However, in the fully adjusted model, the moderating effects of the social capital indicators were able to offset the negative influences of caregiving on health outcomes. More importantly, the research revealed how community level social capital such as community participation, social cohesion and trust realised by individual carers, potentially could mitigate the negative influence of caregiving on health outcomes. This could be driven by the positive and significant influence of social capital on both general and mental health outcomes [20, 43, 44]. Similar to these findings, Oshio and Kan (2016) focussed on social activities involving individual interpersonal interactions with others mainly in their neighbourhood or community (e.g., hobbies or cultural activities, exercise or sports, community events, support for children and the elderly) and concluded that participation in such activities mitigated substantially the negative impact of caregiving on mental health [19].

### Carer health and small area differences

The small area level variations accounted for almost 12% for the total variation in general health, and for almost 13% of the total variation in mental health status of individuals in Australia. In the fully adjusted models, these small area level variations remained statistically significant accounting for about 5% of the total variation in general health and for about 6% of variation in mental health. Additionally, there were statistically significant differences in the magnitude of impact that caregiving exerted on carers’ general and mental health across small areas in Australia. We found a negative association between the magnitude of impact that caregiving had on general health and the average value of general health across SA1s, but a direct positive association between caregiving effects on mental health and average values of mental health at SA1 level.

These findings have important implications for SA1 level policy perspectives with respect to both general and mental health outcomes. For general health, as the average value of general health increases across SA1s in Australia, the impact of caregiving on general health declines (Fig. 2). Therefore, in order to reduce the negative impact of caregiving on general health, policies should be targeted at improving the average general health scores for SA1s. Furthermore, policies aimed at mitigating the negative influence of caregiving on carers’ general health would be more effective in SA1s in the top left quadrant of Fig. 2 than the SA1s in the bottom left quadrant, whereas SA1s in the bottom left quadrant should be targeted for overall improvement in general health scores.

For mental health, as the average value of overall mental health increases across SA1s, the impact of caregiving on mental health increases (Fig. 4). This contrasts with the policy implication for general health at SA1 level. Indeed, increasing the overall mental health scores at SA1 level is not helpful as it does not decrease the negative impact of caregiving on carer mental health. Therefore, policies should rather be targeted to reduce the impact of caregiving on individual carers’ mental health. Furthermore, policies to alleviate negative influence of caregiving on carers’ mental health should be directed to SA1s in the top right quadrant of Fig. 4, whereas SA1s in the bottom left quadrant should be targeted with policies for overall improvement in mental health scores coupled with policies to disentangle the negative impact of caregiving on carer’s health.

These are important small-area level inferences that have not been revealed through any previous research examining the influence of caregiving on the general and mental health status of individuals in Australia. Identifying geographical variations in health and quantifying their magnitude is a rather new trend in research and policy perspectives. This research moved beyond previous research that looked at the effect of caregiving on carer health in regional and rural areas [2, 3]. While previous studies found that carers in regional and rural areas suffered worse health outcomes than their urban counterparts, due to remoteness and distances to services, this study shows SA1 level variations, irrespective of rurality, in the impact of caregiving on both general and mental health and drew policy implications. This is really a move forward in promoting policies to identify small area level variations and devise systemic policy changes that would have rather a bottom-up than a top-down approach to implementation.

### Social capital and Carer health

An almost 7% decrease in the variations in both health component scores attributed to small areas were accounted for by the inclusion of individual level demographic, economic and health behaviour related contextual factors and social capital variables in the fully specified models. More importantly, the inclusion of social capital variables that represented societal level social capital - community participation, social cohesion and trust on the local community, realised or harnessed at individual level, considerably reduced the magnitude of negative impact of caregiving on carers’ health. Furthermore, these individual level societal social capital variables, once interacted with carer status in the fully adjusted models, the consequent significant moderation effects alleviated the negative impacts of caregiving on both health components scores. This is an indication that interventions to improve societal social capital components and encouraging carers to harness more of those resources, would deliver robust health outcomes for them.

#### Community participation

Community participation included civic engagement, political participation and breadth of participation as one component and informal social connectedness as another component in this study ^20 21^. Overall, the civic engagement variable, which represented rather formal communication and involvement at community level, exerted no significant influence on individual level general health, and it had a very small in magnitude negative influence on carers’ general health in the fully adjusted model. However, civic engagement exerted a positively significant influence on individual level mental health but with no specifically significant moderation effect on carers’ mental health. On the contrary, informal social connectedness, which represented rather informal connection with family and friends, exerted a statistically significant influence on individuals’ general health with no specific moderating effect on carers’ health, though it exerted significant influence on non-carers’ general health. However, for mental health, this variable exerted significant positive influence on all individuals with significant moderating effect on carers’ mental health. It appeared that for carers informal community participation was helpful than somewhat formal commitments. This may be due to the circumstances that formal commitments may limit the time and resources available for their caregiving responsibility.

#### Social cohesion

Previous studies examined the concept of social cohesion and investigated the relationship between social cohesion and individual health [45]. The literature on social cohesion converges on the view that social cohesion is not a homogenous concept. It includes social justice, social relationships and social exclusion as different dimensions. Much of the debate on social cohesion, in recent days, focuses on social exclusion [45, 46]. Social cohesion was included in this study in a holistic sense as personal social cohesion and personal social exclusion are two separate components. This is based on the belief that these two components capture specific dimensions of social cohesion independently, [47] rather than exerting mutually opposing influences on the individuals’ health. These two social cohesion variables exhibited significant influences (in the expected directions) on individual level general and mental health scores with even significant effect modifications. These variables represented the extent of social support and social isolation of the individual within the community and proved helpful in reducing the negative influence of caregiving on carer’s health.

#### Trust and reciprocity

There is a growing literature with the view of trust as a foundation of social orders [48, 49]. Likewise, components of societal trust and reciprocity were included in this study as levels of trust and distrust on the community. This view is supported by literature that we need to understand both trust and distrust if we are to understand the different ways how trust works ^48 50^. They do not really exert opposing influences and distrust is not merely the absence of trust. Like trust, distrust has its own normative dimension [48, 50]. These two variables simultaneously represented levels of trust and distrust within relational framework based on assumptions of multidimensionality in relationships [49]. These variables came up as significant influences (in the expected directions) for both general and mental health of individuals in this study, with specific moderating effects for carers [51]. They were helpful in reducing the negative influence of caregiving on carers’ health. Since they reflect levels of trust and reciprocity in the community perceived at individual level, they may serve as the foundation of overall personal social capital.

### Strengths and limitations

To sum up, this study has added value to the literature by identifying the moderating impact of social capital in offsetting the negative impacts of caregiving on carers’ health outcomes. The study also highlighted that the negative impacts of caregiving significantly vary in nature and magnitude across small areas in Australia. It is evident that factors at the community level are important. So, policies targeting to improve social capital and carers’ health in terms of promoting community participation, social cohesion, trust and reciprocity need to have a small area, i.e. local, focus rather than taking a ‘one-size-fits-all-regions’ approach.

The strength of this study lies in the inclusion of a large set of nationally representative relevant and potentially influencing variables and using advanced multi-level mixed effects regression modelling to an existing cross-sectional data set (wave 14 of HILDA). While HILDA is longitudinal in nature, we have used the latest wave containing information on community participation, social cohesion, trust and reciprocity. The multi-level mixed effects technique allowed us to model the hierarchical structure of the data set where individual carers/non-carers were nested within small geographical areas where policies and more importantly social capital and area context might vary. Using multi-level mixed effects modelling, we have been able to tease out and quantify the small area level variations in average levels of health scores and in the effects of caregiving on health scores across Australia.

On the other hand, the first weakness of the study lies in using an observational data set as opposed to a data set from a controlled randomized design, and the nature of the cross-sectional data as opposed to the longitudinal data. Secondly, given the nature of HILDA surveys, certain groups of the Australian population such as immigrants, people from culturally and linguistically diverse (CALD) communities may have been inadequately covered in the survey. Thirdly, there may still be a significant portion of the variation in health outcomes associated with unobserved small area contextual variables independently of individual attributes or with unobserved individual level attributes independently of small area indicators that are not accounted for in this analysis. Furthermore, the manner in which this study investigated how the nature/attributes of the local community and community social capital experienced at the individual level moderated carer’s health it fails to account for the possibility that caregiving status and other individual attributes (e.g., personality traits) may in turn affect individuals’ perception of their community and the inherent social capital and other area-related attributes.

## Conclusion

This study makes a significant contribution to the carer health literature in Australia. While there has been previous evidence that caregiving has a negative influence on carers’ general and mental health status, the finding of this study helps in understanding how the negative impact of caregiving on carers’ health varies across small areas in Australia. More importantly, carers’ social capital in varying forms proved to be important for offsetting the negative health effect of caregiving on carer health. Combining these two findings, the study makes significant inferences for designing interventions and small area level policy perspective to improve overall general and mental health status of carers and reduce the impact of caregiving on carer health. Policy interventions designed to develop carer social capital could support informal carers in terms of reducing the impact of caregiving on their health. At the same time, our study suggests that customizing interventions at a local area level (in this case SA1) might optimise return on the investment of public funds.

## Supplementary Information

**Additional file 1.**

## Data Availability

This paper uses unit record data from the Household, Income and Labour Dynamics in Australia (HILDA) Survey. The HILDA survey data is one of the Australian Government Department of Social Services (DSS) longitudinal datasets housed by the National Centre for Longitudinal Data (NCLD) and managed by the Australian Data Archive (ADA). The datasets analysed and/or generated during the current study are subject to the Confidentiality Deed signed with the Commonwealth of Australia (as represented by the Department of Social Services) and to the Commonwealth privacy laws. Data are accessible from the NCLD by application (https://www.dss.gov.au/national-centre-for- longitudinal-data-ncld/access-to-dss-longitudinal-datasets), and any questions about applying for the DSS longitudinal datasets should be addressed to NCLD (ncld@dss.gov.au).

## Abbreviations

ASGS: Australian Statistical Geography Standard
CALD: Culturally and Linguistically Diverse
HILDA: Household Income and Labour Dynamics of Australia
SA1: Statistical Area Level 1
SA2: Statistical Area Level 2
SCQ: Self-completion Questionnaire
SF-36: Short Form 36 Questionnaire
