# Supplementary material for: A multilevel mixed effects analysis of informal carers health in Australia: the role of community participation, social support and trust at small area level
Source: BMC Public Health. 2020 Nov 26;20:1801. doi: 10.1186/s12889-020-09874-0 (PMC7690182; doi:10.1186/s12889-020-09874-0)
Supplement: Supplementary file 1 — Additional file 1. [file 12889_2020_9874_MOESM1_ESM.docx]

## **Additional File:**

**Table A1:** **Community Participation:** **Rotated factor loadings (pattern matrix) and unique variances.**

| Variable (n= 37290) | Civic engagement, political participation and breadth of participation | Informal social connectedness | Unique Variation in Variables |
| --- | --- | --- | --- |
| Community participation: Attend events that bring people together such as fetes, shows, festivals or other community events | 0.505 | 0.473 | 0.521 |
| Community participation: Get involved in activities for a union, political party, or group that is for or against something | 0.756 | 0.031 | 0.428 |
| Community participation: Make time to attend services at a place of worship | 0.590 | 0.078 | 0.645 |
| Community participation: Encourage others to get involved with a group that’s trying to make a difference in the community | 0.841 | 0.141 | 0.273 |
| Community participation: Volunteer your spare time to work on boards or organising committees of clubs, community groups or other non-profit organisations | 0.755 | 0.150 | 0.407 |
| Community participation: Get in touch with a local politician or councillor about issues that concern me | 0.762 | 0.021 | 0.419 |
| Community participation: Give money to charity if asked | 0.406 | 0.314 | 0.736 |
| Community participation: Have telephone, email or mail contact with friends or relatives not living with you | -0.005 | 0.804 | 0.353 |
| Community participation: Chat with your neighbours | 0.296 | 0.462 | 0.699 |
| Community participation: Talk about current affairs with friends, family or neighbours | 0.348 | 0.515 | 0.613 |
| Community participation: Make time to keep in touch with friends | 0.087 | 0.794 | 0.362 |
| Community participation: See members of my extended family (or relatives not living with me) in person | 0.117 | 0.654 | 0.559 |

**Table A2: Personal Social Cohesion: Rotated factor loadings (pattern matrix) and unique variances.**

| Variable (n= 184076) | Personal Social Exclusion | Personal Social Cohesion | Unique Variation in Variables |
| --- | --- | --- | --- |
| People don’t come to visit me as often as I would like | 0.728 | 0.015 | 0.469 |
| I don’t have anyone that I can confide in | 0.750 | -0.360 | 0.308 |
| I have no one to lean on in times of trouble | 0.760 | -0.367 | 0.288 |
| I often feel very lonely | 0.729 | -0.236 | 0.412 |
| I often need help from other people but can’t get it | 0.766 | -0.182 | 0.380 |
| When I need someone to help me out, I can usually find someone | -0.411 | 0.759 | 0.254 |
| I enjoy the time I spend with the people who are important to me | -0.146 | 0.825 | 0.298 |
| There is someone who can always cheer me up when I am down | -0.291 | 0.703 | 0.421 |
| When somethings on my mind, just talking with the people I know can make me feel better | -0.102 | 0.861 | 0.248 |
| I seem to have a lot of friends | -0.354 | 0.451 | 0.671 |

**Table A3: Trust and Reciprocity: Rotated factor loadings (pattern matrix) and unique variances.**

| Variable | Trust | Distrust | Unique Variation in Variables |
| --- | --- | --- | --- |
| Most people you meet keep their word | 0.79 3 | -0.254 | 0.307 |
| Most people you meet make agreements honestly | 0.858 | -0.204 | 0.222 |
| Generally speaking, most people can be trusted | 0.821 | -0.215 | 0.280 |
| Most of the time people try to be helpful | 0.855 | -0.168 | 0.241 |
| Most people would try to take advantage of you if they got a chance | -0.294 | 0.755 | 0.343 |
| Most people you meet succeed by stepping on other people | -0.356 | 0.749 | 0.312 |
| People mostly look out for themselves | -0.079 | 0.803 | 0.349 |
